# The Mitochondrial Genome of an Aquatic Plant, *Spirodela polyrhiza*

**DOI:** 10.1371/journal.pone.0046747

**Published:** 2012-10-04

**Authors:** Wenqin Wang, Yongrui Wu, Joachim Messing

**Affiliations:** Waksman Institute of Microbiology, Rutgers, The State University of New Jersey, Piscataway, New Jersey, United States of America; University of Connecticut, United States of America

## Abstract

**Background:**

*Spirodela polyrhiza* is a species of the order Alismatales, which represent the basal lineage of monocots with more ancestral features than the Poales. Its complete sequence of the mitochondrial (mt) genome could provide clues for the understanding of the evolution of mt genomes in plant.

**Methods:**

*Spirodela polyrhiza* mt genome was sequenced from total genomic DNA without physical separation of chloroplast and nuclear DNA using the SOLiD platform. Using a genome copy number sensitive assembly algorithm, the mt genome was successfully assembled. Gap closure and accuracy was determined with PCR products sequenced with the dideoxy method.

**Conclusions:**

This is the most compact monocot mitochondrial genome with 228,493 bp. A total of 57 genes encode 35 known proteins, 3 ribosomal RNAs, and 19 tRNAs that recognize 15 amino acids. There are about 600 RNA editing sites predicted and three lineage specific protein-coding-gene losses. The mitochondrial genes, pseudogenes, and other hypothetical genes (ORFs) cover 71,783 bp (31.0%) of the genome. Imported plastid DNA accounts for an additional 9,295 bp (4.1%) of the mitochondrial DNA. Absence of transposable element sequences suggests that very few nuclear sequences have migrated into *Spirodela* mtDNA. Phylogenetic analysis of conserved protein-coding genes suggests that *Spirodela* shares the common ancestor with other monocots, but there is no obvious synteny between *Spirodela* and rice mtDNAs. After eliminating genes, introns, ORFs, and plastid-derived DNA, nearly four-fifths of the *Spirodela* mitochondrial genome is of unknown origin and function. Although it contains a similar chloroplast DNA content and range of RNA editing as other monocots, it is void of nuclear insertions, active gene loss, and comprises large regions of sequences of unknown origin in non-coding regions. Moreover, the lack of synteny with known mitochondrial genomic sequences shed new light on the early evolution of monocot mitochondrial genomes.

## Introduction

Usually, a plant cell contains three genomes: plastid, mitochondrial, and nuclear. In a typical *Arabidopsis* leaf cell, there are about 100 copies of mitochondrial DNA (mtDNA), about 1,000 copies of chloroplast DNA (cpDNA), and two copies of nuclear DNA (ncDNA) [1].

The mitochondrial genome plays fundamental roles in development and metabolism as the major ATP production center via oxidative phosphorylation [2]. The mitochondrial genetic system in flowering plants exhibit multiple characteristics that distinguish them from other eukaryotes: large genome size with dispersed genes, an incomplete set of tRNAs, trans-splicing, and frequent uptake of plastid DNA or of foreign DNA fragments by horizontal and intracellular gene transfer [2], [3], [4], [5], [6]. Plant mtDNAs are a major resource for evolutionary studies, because coding regions evolve slowly, in contrast to the flexible non-coding DNA. Therefore, the structural evolution and plasticity of plant mtDNAs make them powerful model for exploring the forces that affect their divergence and recombination.

With the emergence of second-generation sequencing technologies, the number of completed plant mitochondrial genomes deposited in the GenBank database (http://www.ncbi.nlm.nih.gov/genomes/GenomesGroup.cgi?taxid=33090&opt=organelle. Accessed 2012 Sep 11) has increased until August of 2012 to 69. Most are from Chlorophyta (17 of green algae) and seed plants (26 of eudicotyledons). So far, among 11 sequenced monocot mt genomes, 10 are from the Poales, which have been extensively studied and only one, *Phoenix*, a palm, from the order of Arecale has been sequenced [7]. Obviously, complete mt sequence data will be needed not only from closely but also distant related taxa to give us a broader perspective of mt genome organization and evolution.

*Spirodela polyrhiza*, with great potential for industrial and environmental applications, is a small, fast growing aquatic plant in the Araceae family of the Alismatales order [8], [9]. There are 14 families, 166 genera, and about 4,500 species in this order. The early diverging phylogenetic position of Alismatales offers a broader view at features of monocot mt genomes. Plant mitochondria could also open a strategy for transgenes with high expression level and biological containment because of their maternal inheritance [10]. Here, we demonstrate the *de novo* assembly of a complete mt genome sequence from total leaf DNA using the SOLiD sequencing platform and a genome copy number-sensitive algorithm that can filter chloroplast and nuclear sequences. Indeed, comparative analysis of this genome provides us with unique features and new insights of this class of plants that differ from other monocots.

## Materials and Methods

### DNA Isolation and SOLiD DNA Sequencing

The methods for DNA extraction and DNA sequencing by the SOLiD platform followed a protocol as previously published [11]. Briefly, total genomic DNA was extracted from the clonally grown whole plant tissue of *Spirodela polyrhiza*. A mate-paired library was made with 1.5 Kb insertions and read length was 50 bp. Since nucleic, mitochondrial and chloroplast sequence all exist in reads from total DNA preparation, copy number between three genomes was significantly different [12], [13], so that it was feasible to *de novo* assembly both chloroplast and mitochondria genomes using the same dataset but with different coverage cut-off numbers as described previously [11].

### Genome Assembly, Finishing and Validation

**Figure 1:**
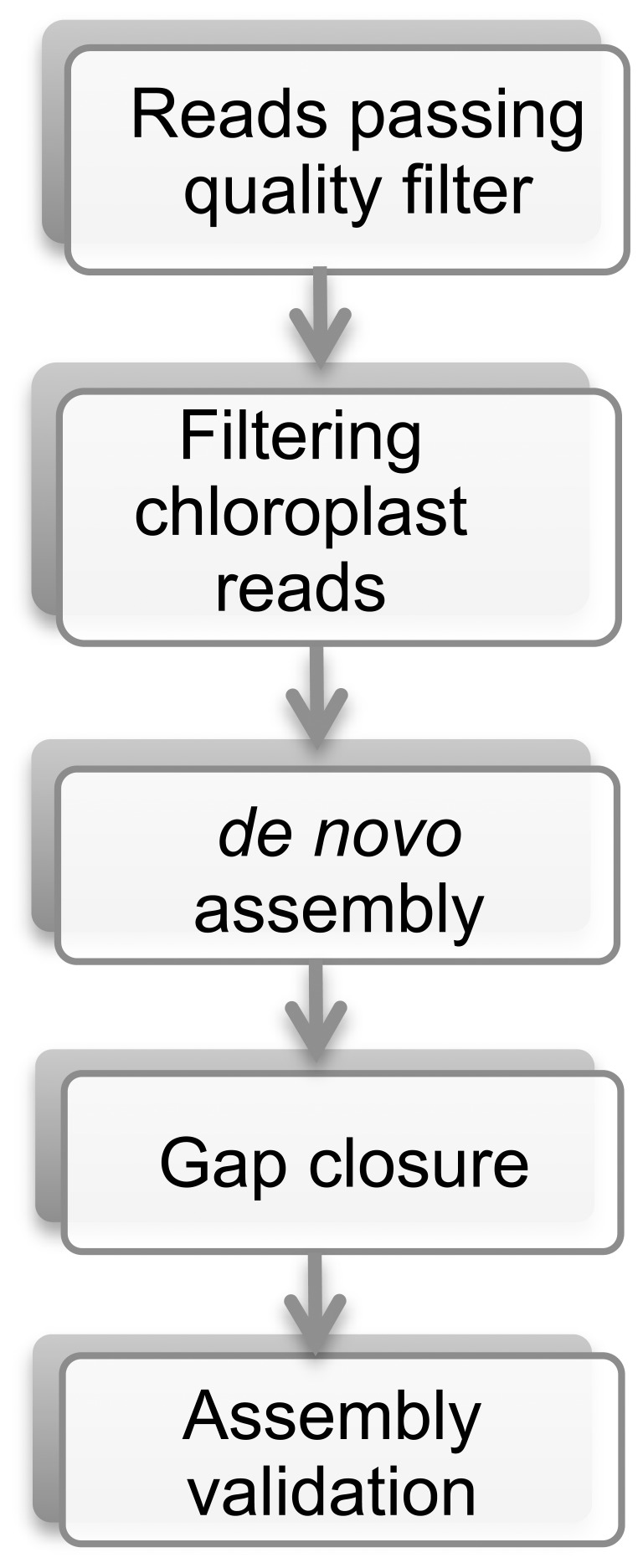
Pipeline of mitochondrial genome assembly. Details were described in Methods.

The coverage cut-off was fully utilized to only allow the target organellar genome to be assembled due to obvious differentiation of copy number for three genomes in total reads [12]. Furthermore, low-level contaminating sequences from foreign DNA (mainly nuclear DNA) were discarded by this approach. Quality control and other details were described recently [11]. Before we assembled the mitochondria genome using mate-paired reads, we masked chloroplast reads to reduce effects due to plastid sequence predominance. The detailed pipeline was shown below (Fig. 1).

1) Filtering chloroplast reads: we mapped total high quality reads to existing chloroplast genome (GenBank # JN160603) by BWA short-read alignment component with default parameters [14]. Only unmapped reads were used in the next step. 2) *de novo* assembly: the assembly was executed using the SOLiD™ System *de novo* Accessory Tools 2.0 (http://solidsoftwaretools.com/gf/project/denovo/) in conjunction with the Velvet assembly engine [15]. 3) Gap closure: since chloroplast reads were pre-removed before mitochondrial assembly, theoretically, any location with chloroplast insertion in mtDNA would create a gap. Using flanking primers bridging 57 gaps, the missing sequences were amplified and sequenced with the ABI 3730×l system, yielding a complete contiguous mtDNA sequence (Table S1). To validate the circularity of the *Spirodela* mtDNA, PCR products were sequenced with pairs of primers bridging gaps and overlapping with the assembled linear scaffold. 4) Most gaps were small enough for single CE (capillary electrophoresis) sequence reads and overlapping sequences served as a measure for the accuracy of the SOLiD assembly and error rate. Therefore, PCR amplification and CE sequence provided validation of the order of contigs and also revealed sequencing discrepancies between these two platforms.

### Genome Annotation and Sequence Analysis

The main pipeline for mitochondrial genome annotation was adapted from other sources [5]. Databases for protein-coding genes, rRNA and tRNA genes were compiled from all previously sequenced seed plant mitochondrial genomes. BLASTX and tRNAscan-SE were the mainly used programs [5]. The boundaries for each gene were manually curated. The sequin file including sequence and annotation was submitted to NCBI GenBank as JQ804980. The graphical gene map was processed by OrganellarGenomeDRAW program [16]. The codon usages for all protein coding genes in *Spirodela* and *Oryza* were calculated by using the Sequence Manipulation Suite [17].

Cp-derived tRNAs were identified by aligning all tRNA in annotated cpDNA to mtDNA with 80% of identity, an e-value of 1e-10 and a 50% coverage threshold. All remaining sequences were further scanned by EMBOSS getorf for open reading frames (ORFs) with more than 300 bp [18].

Putative RNA editing sites in protein-coding genes were identified by the PREP-mt Web-based program based on the evolutionary principle that editing increases protein conservation among species (http://prep.unl.edu/. Accessed 2012 Sep 11) [19]. The optimized cut-off value 0.6 was set in order to achieve the maximal accurate prediction. RNA editing sites from four genes were validated by RT-PCR with gene-specific primers (Table S2).

Sequences transferred to mtDNA were found by BLASTN search of mtDNA against the *Spirodela* chloroplast genome with 80% of identity, e-value of 1e-10 and 50 bp of length threshold. Repeat sequence analysis was predicted by using REPuter web-based interface, including forward, palindromic, reverse and complemented repeats with a cut-off value of 50 bp [20]. The mitochondrial genome was screened by repeatmasker under cross_match search engine (http://www.repeatmasker.org/cgi-bin/WEBRepeatMasker. Accessed 2012 Sep 11) for interspersed repeats and low complexity DNA sequences [21].

### Phylogenetic Analysis

We aligned 19 homologous protein-coding gene sequences (*nad1*, *nad2*, *nad3*, *nad4*, *nad4L*, *nad5*, *nad6*, *nad7*, *nad9*, *cob*, *cox1*, *cox2*, *cox3*, *atp1*, *atp4*, *atp6*, *atp8*, *atp9* and *rps3*) from the *Spirodela* mitochondrial genome and other seven plant organisms (Table S8, *Cycad*, NC_010303; *Phoenix*, NC_016740; *Spirodela*, JQ804980; *Oryza*, NC_011033; *Zea*, NC_007982; *Boea*, NC_016741; *Nicotiana*, NC_006581; *Arabidopsis*, NC_001284) and constructed a phylogenetic tree. Annotations were revalidated and sequences were concatenated into a single continuous sequence from 18,537 to 19,041 bp to initiate alignment by MEGA5 [22]. The phylogeny of the mitochondrial genome was estimated by maximum likelihood (ML) with 1,000 Bootstrap of replicates. *Cycas* was used as the outgroup.

### Comparison of Global Genome Structure

**Figure 2:**
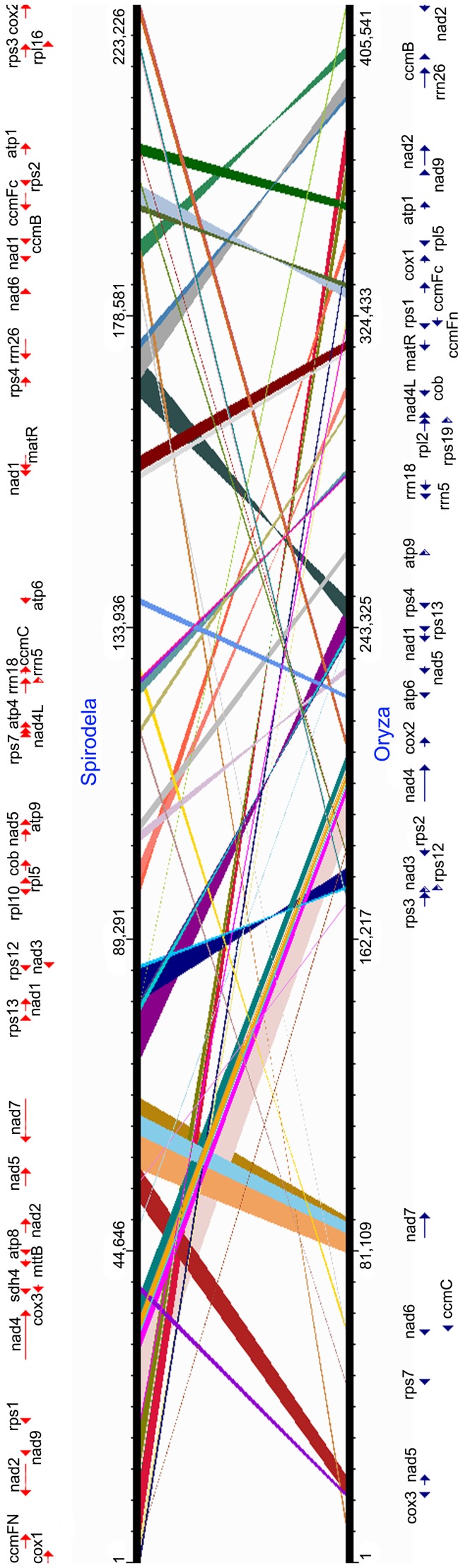
Comparison of synteny in conserved gene loci of *Spirodela* and *Oryza* mitochondrial genomes. The annotated protein-coding genes were indicated for *Spirodela* and *Oryza*. Major conserved regions were bridged by lines. The visualized genome synteny was performed by GSV: a web-based genome synteny viewer [23].

The conserved regions for protein-coding and rRNA genes were identified between *Spirodela* and *Oryza* sequences by BLASTN. The synteny together with the annotation file were uploaded to a web-based genome synteny viewer GSV [23]. The relative ordering of a set of homologous genes was illustrated in Fig. 2.

## Results and Discussion

### The *de novo* Assembly of SOLiD Reads

The optimal parameter of the SOLiD™ System *de novo* Accessory Tools 2.0 for the assembly of the *Spirodela* mitochondrial genome has a hash length of 25 and coverage cut-off of 45. Under these conditions, assembly of SOLiD reads from total leaf DNA resulted in 15 scaffolds and 88 contigs, of which three scaffolds were mitochondrial (173,697, 47,896, 1824 bp) (Table 1). As expected, the other scaffolds were mainly copies of ribosomal RNA genes and retroelements of the nuclear genome because their copy number was comparable to the copies of mitochondrial genomes per leaf cell. To validate the assemblies, gaps were amplified with PCR for dideoxy sequencing with the CE ABI 3730×l system. With this information the order of the three scaffolds were resolved. Furthermore, after the SOLiD short read assembly was aligned with the CE long read sequences, only 0.036% discrepancy was found within 19 Kb sequence of overlaps, demonstrating high consistency between the two platforms. When we mapped the total reads back to the complete mtDNA, a total of 467-fold coverage was calculated. Considering the 5,474-fold chloroplast coverage, we found 41-fold coverage of nuclear genome sequences (Table 1). This level of coverage from assembled sequences was consistent with the expected representation of the three genomes in total leaf DNA, yielding chloroplast, mitochondria, and nuclei with the approximate ratio of 100∶10:1.

**Table 1:** *de novo* assembly statistics for the *Spirodela* mitochondrial genome.

Statistical list	Number
Number of scaffolds	15
N50 scaffolds (bp)	173,697
Number of contigs	88
N50 contigs (bp)	6,528
Sum contig length (bp)	240,987
Hash length	25
Expected coverage	90
Coverage cut-off^a^	45
Total reads (X10∧6)	153
Aligned reads (%)	1.4
Average chloroplast coverage^b^	5,474
Average mitochondrial coverage	467
Average nuclear coverage	41

a Coverage cut-off: minimum coverage required to form a contig.

b Average chloroplast coverage was cited from *Spirodela* chloroplast genome assembly [11].

Here, we applied a layered approach of sequencing organelle genomes without fractionation from total leaf DNA. Thanks to an assembly algorithm of sequence reads that is sensitive to the differential copy number of organelle and nuclear genomes, we did not physically need to fractionate plastid, mitochondrial, and nuclear DNA for deep sequencing. Therefore, we first assembled the complete *Spirodela* chloroplast genome from ABI SOLiD and gap-closure 3730xl reads, which permitted us to mask all plastid DNA reads before assembling mitochondrial DNA, which is in access of nuclear DNA but not as abundant as plastid DNA [11], [24]. Furthermore, we can take advantage of the ratios of these genomes to limit the value of coverage cut-off with identical dataset of SOLiD reads, which is taken in consideration for the assembly algorithm to distinguish between plastid, mitochondrial, and nuclear genome sequence reads [12], [13]. Assemblies were validated like in the case of chloroplast DNA by PCR and gap sequencing of long reads with the traditional ABI 3730×l sequencing system. Following this protocol, we obtained a complete mitochondrial genome from an aquatic plant in a very cost-efficient way, which can serve as a reference for future mt genomics.

### Features of the *Spirodela* Mitochondrial Genome

**Figure 3:**
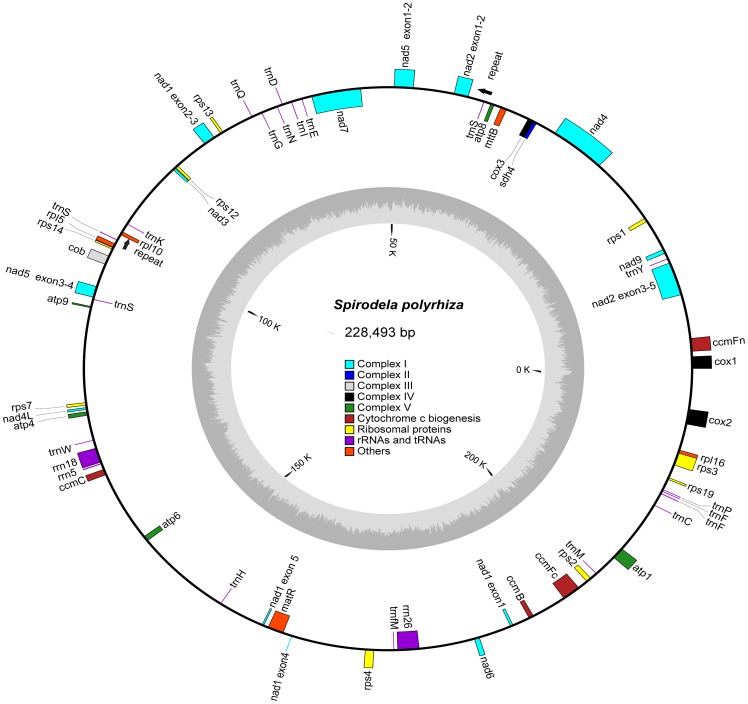
The gene map of *Spirodela polyrhiza* mitochondrial genome. Genes indicated as closed boxes on the outside of the circle are transcribed clockwise, whereas those on the inside were transcribed counter-clockwise. Pseudogenes were indicated with the prefix “Ψ”. The biggest repeat pair was also marked by arrows. The genome coordinate and GC content are shown in the inner circle.

The mitochondrial genome was assembled into a 228,493 bp master circle (Fig. 3), which makes it the smallest genome of all sequenced monocots, much smaller than the 715,001 bp of *Phoenix dactylifera* [7], 490,520 bp of *Oryza sativa* [25], or 569,630 bp of *Zea mays* mitochondria [26]. Because *Spirodela* diverged at a very early stage in the monocot lineage, it suggests that either the common ancestor of monocots had a relatively compact genome, with a series of independent expansions by accumulation of chloroplast and nuclear sequences or proliferation of pairs of repeats, leading to the large genomes in rice and maize [5], [25], [26], or a number of size contractions happened in *Spirodela* from the large genome of their ancestor. The GC content in the mtDNA was 45.7%, slightly higher than 43.8% of *Oryza* and 43.9% of *Zea* [25], [26]. The coding sequences covered 31% of the mitochondrial genome compared with 57.4% of the chloroplast genome [11] (Table 2). There were 57 functional genes and 4 pseudogenes in total, encoding 35 proteins, 19 tRNAs and 3 rRNAs (Table S3). Therefore, it gave rise to a density of 4.0 Kb per gene. Noticeably, eight genes (*ccmFc*, *cox2*, *nad1*, *nad2*, *nad4*, *nad5*, *nad7*, *rps3*) had 15 *cis*-spliced group II introns, whereas *nad1*, *nad2* and *nad5* were disrupted by 6 *trans*-splicing sites (Table 2 and S1). Previous studies suggested that *trans*-splicing had evolved before the emergence of hornworts [27]. In general, the numbers and locations of introns in the *Spirodela* mtDNA were rather well conserved in other sequenced monocot genomes.

**Table 2:** Summary of general features for *Spirodela* mitochondrial genome.

Feature	Value
Genome size (bp)	228,493
GC content (%)	45.7
Coding sequences (%)^a^	31.4
Protein coding gene #	35
ORFs #	39
*cis−/trans-intron #*	15/6
tRNA gene #	19
rRNA gene #	3
Chloroplast-derived (%)	4.1
Gene density (bp)	4009

a coding sequences include identified mitochondrial genes, pseudogenes, ORFs and *cis*-spliced introns.

### Protein Genes and Transcript Editing

The content of key protein coding genes in *Spirodela* mtDNA is highly conserved with other angiosperms [26], [28], [29], [30]. There were nine subunits of the oxidative phosphorylation complex I (*nad1*, 2, 3, 4, 4L, 5, 6, 7 and 9); one subunit of complex II (*sdh4*); one subunit of complex III (*cob*); three subunits of complex IV (*cox1*, *cox2* and *cox3*); five subunits of complex V (*atp1*, 4, 6, 8 and 9); and four subunits of a complex involved in cytochrome c biogenesis (*ccmB*, *ccmC*, *ccmFn* and *ccmFc*). Other genes encoding maturase (*matR*) and transport membrane protein (*mttB*) were also present in *Spirodela* mtDNA. As in maize [26], the matR gene in *Spirodela* also resided in the intron 4 of *nad1*, which is trans-spliced after transcription. In *Spirodela*, there were ten functional ribosomal genes and two pseudogenes of *rps14* and *rps19* with early stop codons, whereas rice had a functional *rps19* and a non-functional *rps14* [25] and both were missing in maize (Table S3) [26]. All annotated genes and coordinates were listed in Table S3 and shown in a graphical map (Fig. 3).

Post-transcriptional editing occurs in nearly all plant mitochondria, which results in altered amino acid sequences of the translated protein by converting specific Cs into Us in their transcripts. We used the program of the predictive RNA editor of plant mitochondrial genomes (PREP-mt) to predict the location of RNA editing sites, which are based on well-known principles that plant organelles maintain the conservation of protein sequences across many species by editing mRNA [19]. By setting the cut-off value to 0.6 within the 35 protein-coding genes of *Spirodela* mtDNA 600 sites were predicted as C-to-U RNA editing sites (Table S4). To validate the accuracy of this prediction, we compared RNA transcripts from *atp9*, *nad9*, *cox3* and *rps12* by RT-PCR with the corresponding genomic sequences yielding a confirmation for 90.8% of the predicted sites. Considering a level of about 10% artificial predictions, we estimate about 540 RNA editing sites, a number that lies between the 441 of protein-coding genes of *Oryza* [25] and 1,084 of *Cycas* [29].

It is generally accepted that RNA editing is essential for functional protein expression as it is required to modify amino acids to maintain appropriate structure and function [31], or to generate new start or stop codons [32]. Indeed, the abundance of RNA editing sites in *Spirodela* mtDNA might have increased genome complexity and pace of divergence. We summarized the number of potentially modified codons of *Spirodela* mtDNA in Table S5. Three edited codons (TCA (S) = >TTA (L); TCT (S) = >TTT (F); CCA (P) = >CTA (L)) were found most frequently, whereas three editing events from two codons (CAA (Q) = >TAA (X); CAG (Q) = >TAG (X)) resulted in stop codons (Table S5). Even though three new stop codons are located close at the carboxyterminal end of proteins (ccmC, rps1 and rpl16), it is not clear whether these small truncations affect their functions or not, which would require experimental evidence.

### The rRNA, tRNA Genes and Codon Usage

**Figure 4:**
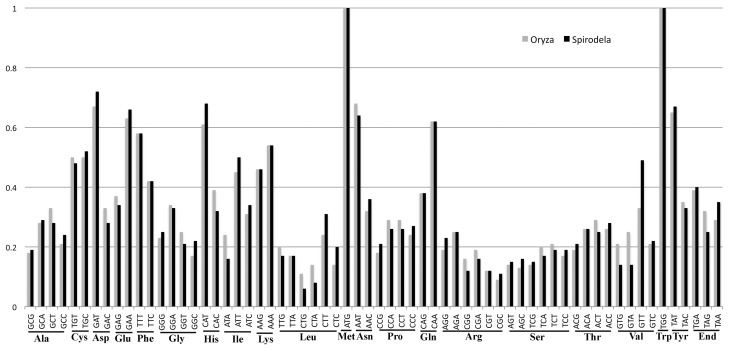
The fraction of each codon usage among the same amino acid in *Spirodela* compared to that in *Oryza*. Black bar was *Spirodela* and grey was *Oryza*. The fraction of each codon usage was shown on Y-axis.

*Spirodela* mtDNA contains 3 ribosomal RNA genes (*rrn5*, *rrn18*, *rrn26*) and one pseudogene of *rrn26*. The 19 putatively expressed tRNA genes are specific for 15 amino acids (Table S3). Four of them (*trnN*-GTT, *trnH*-GTG, *trnM*-CAT and *trnS*-GGA) are probably chloroplast-derived because of high sequence similarity. They are also predicted as chloroplast origin in maize, rice, sugar beet and *Arabidopsis* except *trnS*-GGA in maize [26]. Therefore, they were not recently acquired from chloroplast, but more likely an event of horizontal transfer in a common ancestor. One *trnH*-GTG is considered to be a non-functional pseudogene. Functional tRNA genes for the amino acids Ala, Arg, Leu, Thr and Val are absent. Because all 20 amino acids are required for protein synthesis, and all 64 codons are used in the *Spirodela* mt genome based on a codon-usage scan (Fig. 4 and Table S6) [17], the missing tRNAs are presumably encoded by the nuclear genome and imported from the cytosol into the mitochondria [33]–[34]. We also found that the two codons for TAT-Tyr and TTT-Phe are highly preferred in *Spirodela* and *Oryza* and overall other codon usage is rather similar between the two species (Table S6).

### ORFs and Intergenic Sequences

Only ORFs encoded by a hypothetical gene with more than 300 bp in length between start and stop codons and no match with a known mt coding sequence were counted. Based on this cut-off, we found 39 mitochondrial ORFs, most of which were not cp migrations and specific to *Spirodela* (Table S3). We named ORFs using their amino acid numbers. When the same length of ORFs happened, a lower case letter (a, b, c, etc) was added. Given the large amount of intergenic DNA in *Spirodela* mtDNA, it is not surprising to find an abundance of additional ORFs in its genome. Rarely, ORFs showed conservation to any other plants so that putative ORFs were considered to be spurious prediction [35]. However, orf100a had an ortholog of a NADH-ubiquinone oxidoreductase chain in *Nicotiana tabacum* (GenBank: YP_717128) and orf257 had sequence similarity to DNA polymerase (GenBank: YP_003875487) found in plant mt plasmids [4]. Some studies found that unidentified ORFs had transcripts in rapeseed [36] or to be actively transcribed in sugar beet [37], but further studies are needed to determine whether they encode functional proteins.

A striking feature of *Spirodela* mtDNA was that 81% of the intergenic regions were species-specific and showed no sequence similarity to any other known sequence. It seemed that anonymous sequences in intergenic DNA were quite common. For instance, unidentifiable sequences comprised 70% of *Beta vulgaris* mtDNA [38]. Although they split about 50 million years ago, 76% of rice mtDNA sequences appeared to be highly divergent from maize in intergenic regions [26]. The repetitive DNAs [39], mt plastidal migrations [40] and viral DNA insertions [41] could contribute to the expansion of intergenic regions, but still comprised a rather small fraction in most seed plant mt genomes. On the other hand, it was quite common that multipartite mt genomes could be generated through large repeat pairs with high frequency [35]. Indeed, 29 potential candidates of repeat pairs with more than 50 bp were found in *Spirodela* mtDNA by using REPuter [20] (Table S7). However, we could not detect repeat-specific contigs from the assembly that could be explained of isomeric and subgenomic molecules derived from a master circle after recombination. Probably, the high rate of non-coding sequence turnover in *Spirodela* mtDNA was mainly generated through the process of micro-homologous recombination or non-homologous end joining, later on of active rearrangement and continuous reshuffling. Still, the high proportion of enigmatic non-coding regions in mtDNA is quite extensive. To understand where all these enigmatic sequences might come from and why they appeared to be so common would require additional sequences from closely related species.

### Phylogenetic Analysis and Gene Loss in Angiosperm Mitochondrial Genomes

**Figure 5:**
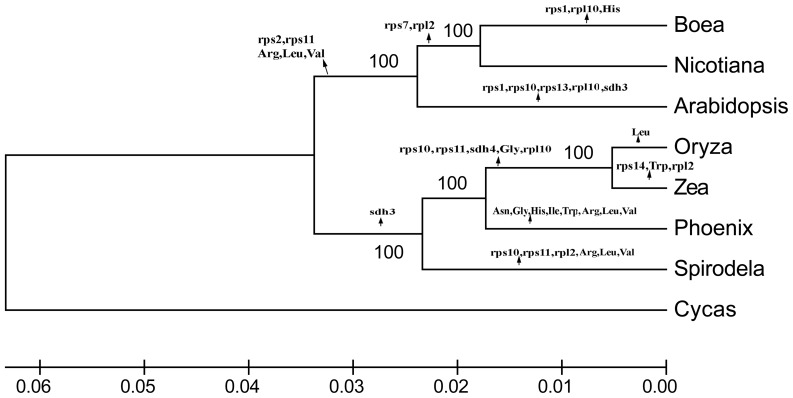
Phylogenetic tree based on 19 conserved genes in mitochondrial genomes. The ML calculation was run by MEGA5 with 1,000 bootstrap replicates. All the gene losses were mapped on the tree branches. *Cycas* was included in the analysis as an outgroup. The signs of Amino Acid (Ala, Arg, Leu, Thr, His, Trp, Ile, Gly, Leu and Val) mean corresponding functional tRNA genes were absent in their mtDNAs.

After re-examining mitochondrial genome annotations from seven species, a selection of 19 conserved genes (*nad1*, *nad2*, *nad3*, *nad4*, *nad4L*, *nad5*, *nad6*, *nad7*, *nad9*, *cob*, *cox1*, *cox2*, *cox3*, *atp1*, *atp4*, *atp6*, *atp8*, *atp9* and *rps3*) was concatenated to permit alignment analysis of 19,824 sites in eight genomes, listed in Table S8 (dicot: *Arabidopsis*, *Nicotiana* and *Boea*; monocot: *Spirodela*, *Phoenix*, *Oryza* and *Zea*; outgroup: *Cycas*). The gene tree topology from multiple loci (Fig. 5) was largely congruent with the known phylogenetic relationships inferred from analysis of rbcL. There were two subclades of monocots and dicots within the angiosperm [42]. Previous studies of fossil records [43], morphology and molecular analysis [44] also supported that Alismatales (*Spirodela*) was a basal monocot followed by Arecales (*Phoenix*), whereas the Poales (rice and maize) resided in the most developed positions.

The loss of protein coding and tRNA genes in seven genomes relative to the outgroup was examined based on the phylogenetic tree. Generally, most losses were limited in their phylogenetic depth to a single family and must have occurred recently (Fig. 5). Three ribosomal protein genes *rps10*, *rps11* and *rpl2* were missing in *Spirodela* mtDNA. Frequent gene losses of ribosomal protein genes also occurred in other species. At a closer look, *rps2* seemed to have been lost early in the evolution of dicots, whereas *rps2* was present in *Cycas*, *Marchantia*, and other monocots [45]. The *rps11* gene was missing in dicots (*Arabidopsis*, *Nicotiana* and *Boea*) and also in some monocots (*Spirodela, Oryza* and *Zea*). The corresponding mt *rps2* and *rps11* genes have been transferred to the nucleus in *Arabidopsis*, soybean, and tomato, suggesting that gene loss followed functional transfer to the nucleus [6], [45]. The unparallel loss of *rps11* and *rpl2* in *Spirodela* compared with other monocots suggested that the loss of many genes might have occurred independently in various lineages during speciation of angiosperms. The *sdh3* gene was absent and the *sdh4* gene was present in both *Spirodela* and *Phoenix*, whereas neither was retained in rice and maize (Fig. 5 and Table S8). A previous study showed that *sdh4* losses were concentrated in the monocots and no losses were detected in basal angiosperms by Southern blot survey of 280 angiosperm genera, which further showed most of the losses were limited in phylogenetic depth to a single family [46].

Our data lend support to previous studies that most gene losses occurred with mt ribosomal protein genes and rarely with respiratory genes, which was well documented with a Southern blot survey of gene distribution in 281 diverse angiosperms [6]. When a gene was missing from mtDNA of a given species, it was generally assumed that the original copy had been transferred to the nucleus. Therefore, our results strongly suggested that intracellular gene transfer of ribosomal protein and tRNA genes from mitochondria to the nuclear genome was a frequent process, which in return allowed the nucleus to control the organelle by encoding organelle-destined proteins [33], [34]. Still, functional copies of these putative transferred genes will have to be confirmed after the whole nuclear genome sequence will be available. The finding of many intermediate stages of the *cox2* gene transfer in legumes had shown that physical movement of mtDNA to the nuclear genome was an ongoing process [47].

### Chloroplast DNA Insertions

The *Spirodela* mtDNA contained multiple cp-originated insertions, ranging in size from 69 to 1,048 bp. These sequences added up to 9,295 bp of the total amount of transferred cpDNA (Table S9), accounting for 4.07% of the mtDNA. A total of 4,436 bp was derived from the inverted repeats of the chloroplast genome, whereas 4,859 bp was transferred from single copy regions of cpDNA. The similarity level of each insertion to the chloroplast genome varied between 75% and 100%. Moreover, the migrated plastid fragments had 732 substitutions, 28 insertions, and 49 deletions within 9,295 bp. They also contained fragments of plastid genes, such as *psbA*, *petB*, *psbC* and *ycf1* (Table S9). All of the protein-coding genes of plastid origin in *Spirodela* mtDNA were likely to be non-functional as a result of truncations and mutations, whereas four tRNAs of plastidal origin appeared to be intact. Indeed, chloroplast-derived sequences were very common in plant mt genomes, such as 6% in rice [25], 4% in maize [26] and 1% in *Arabidopsis* [28]. Surprisingly, 42.4% of the chloroplast genome of *Vitis* has been incorporated into its mt genome [48]. And a large segment of 113 Kb from chloroplast sequences was captured by the *Cucurbita* mt genome [5].

### Integrated Nuclear DNA

It is believed that transposable elements in mitochondria are nuclear-derived and are therefore common in mt intergenic regions [38], [49]. For instance, 4% of *Arabidopsis* mtDNA was probably derived from transposons of nuclear origins [28]. Four fragments of transposable elements were found in maize mtDNA [26] and nineteen were identified in rice [25]. However, we could not find any transposons in the *Spirodela* mt genome when we searched against the Repbase repetitive element database [50]. This suggests that either very few nuclear sequences have migrated into *Spirodela* mtDNA or *Spirodela* mitochondria select against transposable elements.

### Comparison of Genome Synteny

A significant degree of synteny was found within mitochondrial genomes of liverworts, mosses, and chlorophytes at the base of land plants, including a set of gene clusters (more than two genes together), such as the ribosomal protein cluster, *ccm* gene cluster, and two regions containing the *nad* and *cox* genes [51]. It was clear that the sequences of protein-coding genes were highly conserved, but the relative order of genes was greatly rearranged between *Spirodela* and rice (Fig. 2). Many ribosomal proteins were independently lost in both *Spirodela* and rice (Fig. 5); therefore, synteny between the remaining genes became harder to detect. The ancestral *cob*-*nad1*-*cox3*-*cox2*-*nad6*-*atp6*-*rps7*-*rps12*-*nad2*-*nad4*-*nad5* gene order of basal land plants has been lost due to various recombination and rearrangement events in angiosperm mtDNA evolution. [4], [41], [52].

In summary, our data provides further evidence that SOLiD platforms can assemble both chloroplast and mitochondrial genomes with regular coverage without any organellar purification (Table 1) [11]. Our analysis of the mt genome of *Spirodela*, having the smallest size among sequenced monocots, elucidates the evolutionary change among monocot mt genomes. Although the critical genes for the electron transport chain in *Spirodela* mtDNA are well conserved, different types of ribosomal protein genes are missing in comparison to other monocots. The number of RNA editing in protein coding genes is within a typical range as other plants. Still, no known transposable elements can be found in its genome, suggesting a rather rare migration from the nucleus to the mitochondria. Sequence-based phylogenetic analysis clearly supports the hypothesis that *Spirodela* is at the very basal lineage of monocots. Comparative analyses of mitochondrial genes between *Spirodela* and rice have shown that the relative order of genes is greatly rearranged over a very short evolutionary time. In this regard, additional complete mitochondrial sequences from closely related species will be needed to fortify the distinct evolution of plant mitochondrial genomes.

## Supporting Information

Table S1 **Primer pairs for gap closure and Sanger sequencing.**
(XLS)

Table S2 **Primer pairs for RNA editing validation by RT-PCR.**
(XLS)

Table S3 **Gene content for Spirodela mitochondrial genome.** Gene content includes protein-coding genes, tRNA, rRNA and putative ORFs. “Ψ” means pseudo gene and “cp-” means chloroplast-derived gene.
(XLS)

Table S4 **Predicted RNA editing numbers in each protein-coding gene for Spirodela mtDNA.** The cutoff value for each predicted site was the percentage of matches in alignment to the corresponding amino acid across species.
(XLS)

Table S5 **Type and number of codon modification in predicted RNA editing sites of Spirodela mtDNA.**
(XLS)

Table S6 **Comparison of codon usage between Spirodela and Oryza.** ^a^Results for 35 protein coding genes in Spirodela with 30,790 bp. ^b^Results for all CDS from Genbank in Oryza with 44,875 bp.
(XLS)

Table S7 **Predicted repeat pairs in Spirodela mtDNA by using REPuter.** “F” and “P” means forward and palindromic matches.
(XLS)

Table S8 **Protein-coding and tRNA gene list in the 8 representative plant mtDNAs.** “+” means present and “–“ means absent.
(XLS)

Table S9 **The regions in Spirodela mtDNA originated from cpDNA with corresponding coordinates and identity.**
(XLS)
